# The effects of the DNA Demethylating reagent, 5-azacytidine on SMCHD1 genomic localization

**DOI:** 10.1186/s12863-020-0809-x

**Published:** 2020-01-15

**Authors:** S. Massah, J. Jubene, F. J. S. Lee, T. V. Beischlag, G. G. Prefontaine

**Affiliations:** 1 Faculty of Health Sciences, Simon Fraser University, 8888 University Drive, Burnaby, BC V5A 1S6 Canada; 2 The Vancouver Prostate Centre, 2660 Oak street, Vancouver, BC V6H 3Z6 Canada

**Keywords:** SMCHD1, DNA methylation, 5-azacytidine, Genomic binding

## Abstract

**Background:**

DNA methylation is an epigenetic modification that mainly repress expression of genes essential during embryogenesis and development. There are key ATPase-dependent enzymes that read or write DNA methylation to remodel chromatin and regulate gene expression. Structural maintenance of chromosome hinge domain containing 1 (SMCHD1) is an architectural protein that regulates expression of numerous genes, some of which are imprinted, that are sensitive to DNA methylation. In addition, SMCHD1 germline mutations lead to developmental diseases; facioscapulohumoral muscular dystrophy (FSHD), bosma arhinia and micropthalmia (BAMS). Current evidence suggests that SMCHD1 functions through maintenance or de novo DNA methylation required for chromatin compaction. However, it is unclear if DNA methylation is also essential for genomic recruitment of SMCHD1 and its role as an architectural protein. We previously isolated SMCHD1 using a methylated DNA region from mouse pituitary growth hormone (*Gh1)* promoter, suggesting that methylation is required for SMCHD1 DNA binding. The goal of this study was to further understand DNA methylation directed role of SMCHD1 in regulating gene expression. Therefore, we profiled SMCHD1 genome wide occupancy in human neuroblastoma SH-SY5Y cells and evaluated if DNA methylation is required for SMCHD1 genomic binding by treating cells with the DNA demethylating reagent, 5-azacytidine (5-azaC).

**Results:**

Our data suggest that the majority of SMCHD1 binding occurs in intron and intergenic regions. Gene ontology analysis of genes associated with SMCHD1 genomic occupancy that is sensitive to 5-azaC treatment suggests SMCHD1 involvement in central nervous system development. The potassium voltage-gated channel subfamily Q member1 (*KCNQ1)* gene that associates with central nervous system is a known SMCHD1 target. We showed SMCHD1 binding to an intronic region of *KCNQ1* that is lost following 5-azaC treatment suggesting DNA methylation facilitated binding of SMCHD1. Indeed, deletion of SMCHD1 by CRISPR- Cas9 increases *KCNQ1* gene expression confirming its role in regulating *KCNQ1* gene expression.

**Conclusion:**

These findings provide novel insights on DNA methylation directed function of SMCHD1 in regulating expression of genes associated with central nervous system development that impact future drug development strategies.

## Background

Structural maintenance of chromosome hinge domain containing 1 (SMCHD1) is a chromatin regulator that adopts a homodimeric arrangement driven by its hinge domain to modify gene expression on the X-chromosome as well as autosomal genes [1–3]. Originally, SMCHD1 was identified in an N-ethyl-N nitrosourea mutagenesis screen as an epigenetic modifier and was suggested to be essential for X-inactivation and survival in females [4]. Later studies confirmed the initial observation and showed that SMCHD1 is essential for methylation of a subset of CpG islands at late stages of X-inactivation [5]. Loss of SMCHD1 is also lethal in male mice in a mixed background, suggesting an essential role for gene regulation on non-sex chromosomes [4, 6]. Indeed, we and others have shown that SMCHD1 is important for regulating monoallelically expressed genes including imprinted genes, and clustered protocadherin genes [1–3]. In humans, SMCHD1 mutations associate with two distinct developmental diseases: FSHD [7, 8] and BAMS [9, 10]. FSHD is a muscular dystrophy affecting upper arm, shoulder and face muscles and is characterized by chromatin relaxation of the D4Z4 microsatellite array on chromosome 4 [11]. The most consistent signature of BAMS individuals is the complete absence of a nose which might accompany other malformations [12]. These findings demonstrate that as an epigenetic modifier, SMCHD1 impacts gene regulation of multiple genomic regions which can result in severe diseases. However, it is still unclear how SMCHD1 works within the epigenetic machinery.

SMCHD1 is a non-canonical member of the SMC protein family [4]. SMCHD1 contains a hinge domain homologous to other members of the SMC family, however, unlike other SMC proteins, SMCHD1 N-terminus encompasses a GHKL (Gyrase, Hsp90, Histidine Kinase, MutL) type ATPase domain [13]. The SMCHD1 hinge domain was suggested to have DNA binding activity and be involved in SMCHD1 homodimerization [14]. Structural studies show that SMCHD1 is likely important for heterochromatin formation over the X chromosome by connecting two chromatin domains enriched for repressive histone marks (H3K9me3 and H3K27me3) [15]. Recent data suggests SMCHD1 plays a role in chromosome conformation and long-range chromatin interaction to regulate gene expression. Previously, we isolated SMCHD1 using methylated DNA and we showed that its binding to the pituitary growth hormone promotor is sensitive to DNA methylation [2]. Therefore, we hypothesized that recruitment of SMCHD1 to the human genome is sensitive to DNA methylation status. Here, we examined whether changes in DNA methylation level induced by a DNA demethylating reagent, 5-azaC, would affect the binding of SMCHD1 over the human genome and its molecular function as a regulatory protein. Thus, using chromatin immunoprecipitation in combination with massively parallel sequencing (ChIP-seq), we obtained information on genomic sites bound by SMCHD1 with high resolution and identified SMCHD1 recruitment sites that are sensitive to DNA methylation using 5-azaC. Here, we show SMCHD1 occupancy is located mostly over intron and intergenic regions and is associated with central nervous system in DNA methylation sensitive manner. SMCHD1 genomic binding coincides with binding sites for transcription factors including beta-beta-alpha-zinc fingers and Helix-loop-Helix families. In addition, we demonstrated DNA methylation sensitive binding of SMCHD1 to an intronic region of *KCNQ1* gene that has a role in central nervous system development. Our study associates the role of an epigenetic regulator with DNA methylation and characterizes its molecular function and downstream action that will have implications for drug development.

## Results

### Identification of genome-wide occupancy of SMCHD1 that is sensitive to 5-azaC

**Fig. 1:**
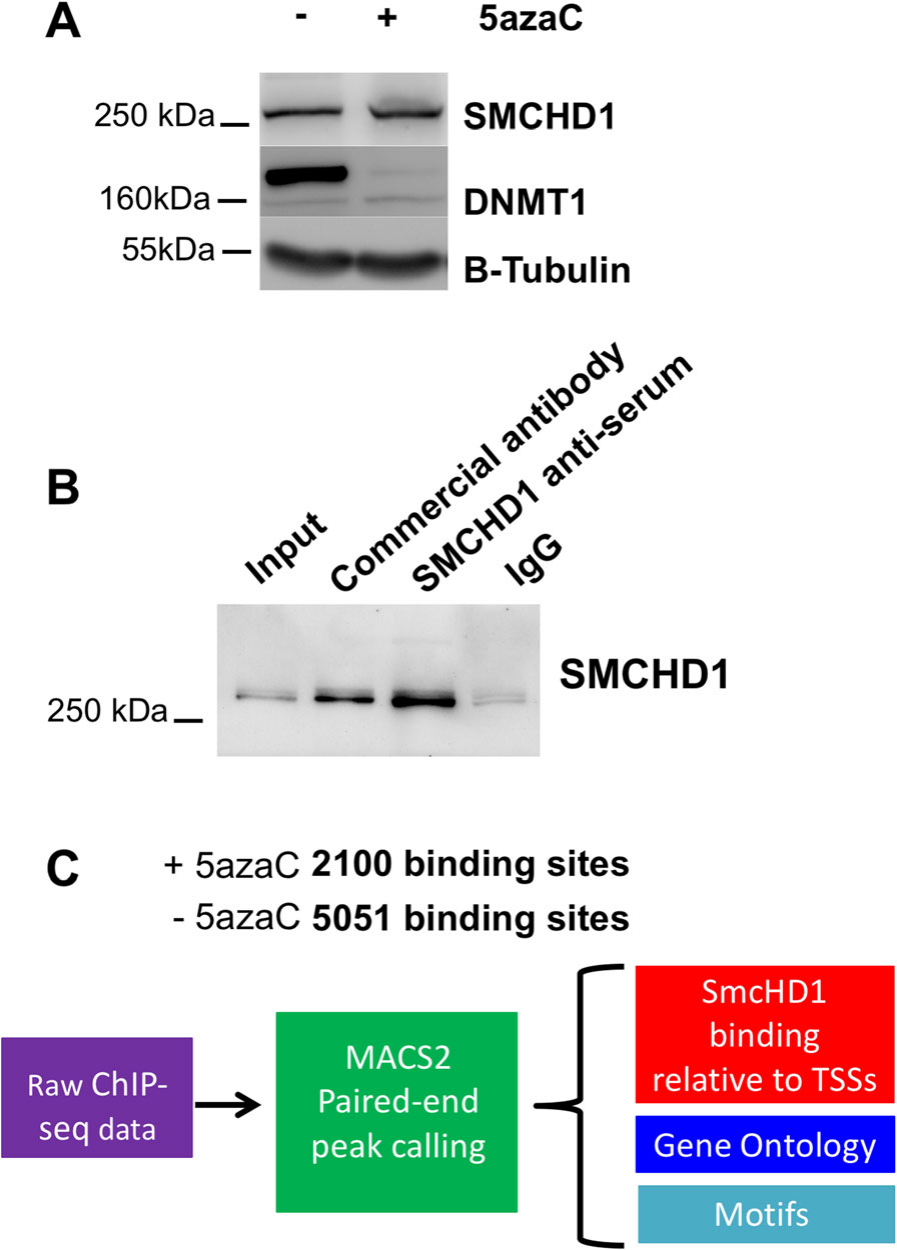
Workflow for SMCHD1 ChIP-seq analysis in SH-SY5Y cells. **a** 5-azaC treatment induces loss of DNMT1 in SH-SY5Y cells while it has no effect on SMCHD1 protein level. β-tubulin was used as an internal control for loading. **b** Immunoprecipitation of SMCHD1 using anti-SMCHD1 antiserum generated in guinea pig, commercial anti-SMCHD1 and guinea pig serum. Immunoprecipitated samples are blotted using anti-SMCHD1 antibody. **c** Schematic illustration of the workflow for analysis of the ChIP-seq data. GREAT algorithm and SeqMonk software determined distribution of SMCHD1 binding sites relative to the associated transcriptional start site (TSS). PAPST and DAVID bioinformatics identified biological processes associated with SMCHD1 occupancy, gene ontogeny, relative to the nearest gene. SeqPos (Galaxy cistrome) identified potential SMCHD1-associated binding motifs

**Fig. 2:**
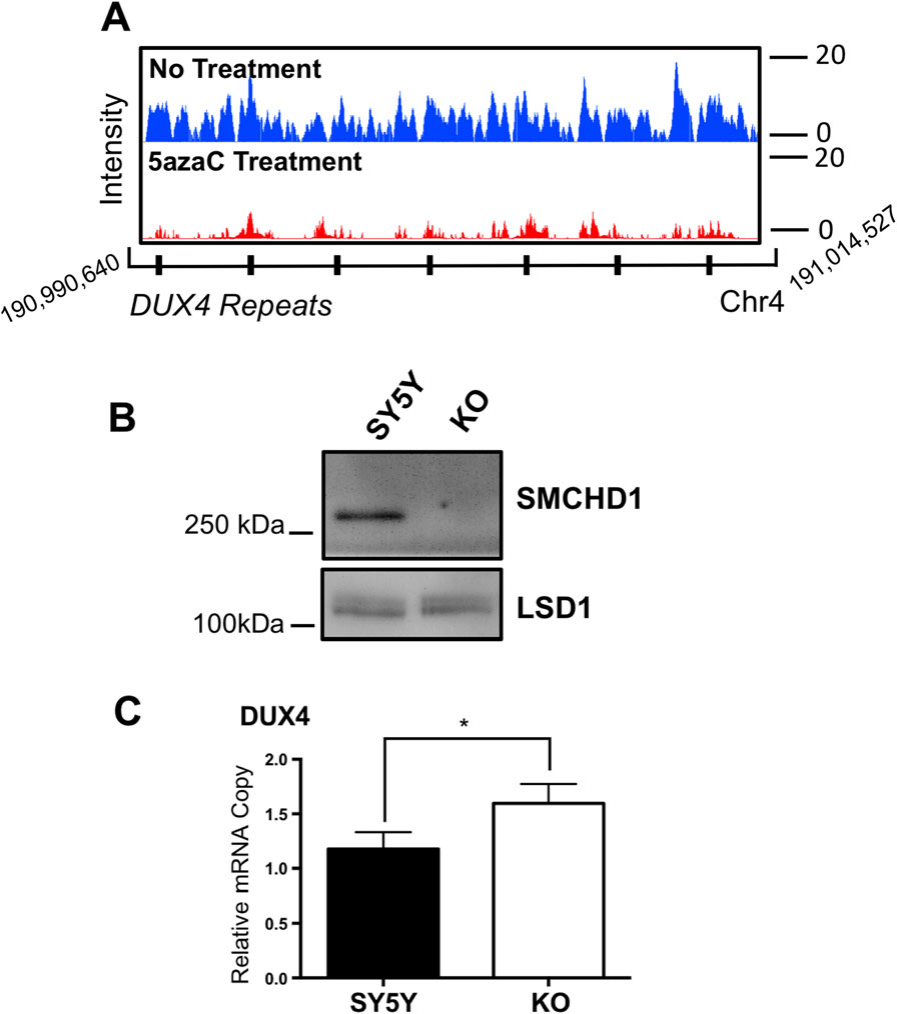
SMCHD1 occupancy over chromatin regions containing *DUX4* gene on chromosome 4. **a** An illustration representing SMCHD1 peaks in SH-SY5Y cells without any treatment (blue) and in cells exposed to 5-azaC (red). **b** Protein levels of SMCHD1 in SH-SY5Y and SMCHD1 sgRNA knockout cells (KO). Lysine specific demethylase 1 (LSD1) was used as an internal control for loading. **c** mRNA quantification of *DUX4* in SH-SY5Y (control) and SMCHD1 KO cells (KO). The copy numbers are relative to beta-actin cDNA levels

Previously, we demonstrated that SMCHD1 DNA binding and DNA methylation were intimately linked [2]. We identified SMCHD1 as a methyl-DNA binding protein using a differentially methylated region located within the mouse pituitary growth hormone (*Gh1*) gene promoter. In addition, we demonstrated that in SH-SY5Y neuroblastoma cells, SMCHD1 regulates expression of imprinted genes associated with two imprinting disorders, Beckwith-Wiedemann and Silver-Russell syndromes (BWS, SRS) [2]. BWS is a growth disorder that is characterized by a number of developmental disorders and embryonal tumors including neuroblastoma [16]. Thus, to investigate DNA methylation dependency of SMCHD1 in regulating gene expression, we sought to compare SMCHD1 genomic localization in SH-SY5Y neuroblastoma cells cultured under normal conditions and those treated with 5-azaC to induce global loss of DNA methylation. In mammals DNA methylation is established mainly by three DNA methyltransferases; DNMT1, DNMT3A and DNMT3B [17, 18]. While DNMT3A and B mediate de novo methylation, DNMT1 is responsible for maintenance and inheritance of DNA methylation after replication [18]. Therefore, loss of DNMT1 protein would significantly affect global levels of DNA methylation. The 5-azaC treatment causes proteolytic degradation of DNMT1, while having low level of toxicity to cells [19], therefore, to examine the efficacy of 5-azaC treatment, we measured DNMT1 protein levels. DNMT1 was almost completely lost in cells treated with 5-azaC while it did not change SMCHD1 protein level (Fig. 1a). We then used equal cell volumes of 5-azaC treated and control SH-SY5Y cells in chromatin immunoprecipitation (ChIP) assays using anti-SMCHD1 antiserum. We generated antiserum in guinea pig specific to hydrophilic antigens of SMCHD1 that efficiently immunoprecipitated SMCHD1 comparable to the commercially available antibody (Fig. 1b). Following chromatin immunoprecipitation, generation of DNA libraries and sequencing, model-based analysis for ChIP-Seq peaks calling with paired ends (MACS2) identified 5051 SMCHD1 binding sites across the genome in cells grown under normal conditions compared to 2100 binding sites in cells treated with 5-azaC (Fig. 1c). In addition, SMCHD1 binding over the previously identified SMCHD1 target gene, *DUX4* gene was greatly affected by 5-azaC treatment and there was a significant reduction in overall SMCHD1 occupation over this region (Fig. 2a). To examine the role of SMCHD1 in regulating *DUX4* gene, using CRISPR-Cas9, we created SMCHD1 knockout SH-SY5Y cells. CRISPR-Cas9 mediated ablation of the *SMCHD1* gene (Fig. 2b) led to significant increase in *DUX4* gene expression as indexed by qPCR (Fig. 2c).

### SMCHD1 binds over intron and intergenic regions and its occupancy associates with central nervous system development

**Fig. 3:**
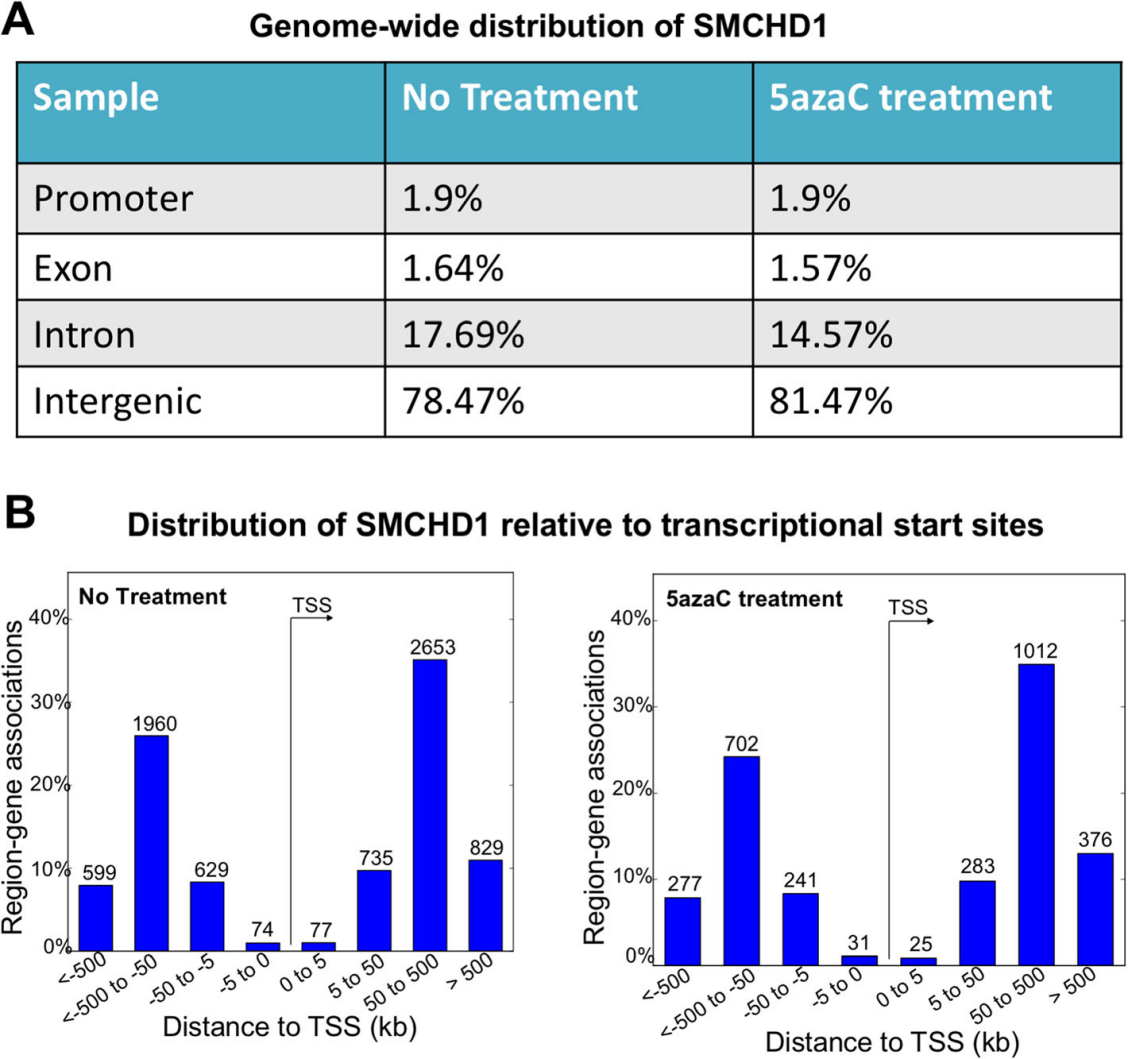
Genome-wide analysis of SMCHD1 binding sites in SH-SY5Y cells. **a** Distribution of SMCHD1 ChIP-seq peaks relative to promoters, gene bodies, exons, introns and intergenic regions. **b** SMCHD1 assigned peaks +/− 500 kb relative to TSS

To elucidate SMCHD1 binding across the genome, we plotted SMCHD1 ChIP-Seq data peaks relative to nearest TSSs. SMCHD1 peaks were mostly found in intergenic and intron regions. When cells were treated with 5-azaC there was a slight redistribution of SMCHD1 binding sites to intergenic regions (from 78.47 to 81.47%) at the expense of those located in introns (from 17.69 to 14.57%) (Fig. 3a). The percentage of SMCHD1 binding sites in exon and promoter regions were unchanged with 5-azaC treatment (Fig. 3a). Most SMCHD1 binding sites were located distal from promoter regions (Fig. 3b).

**Fig. 4:**
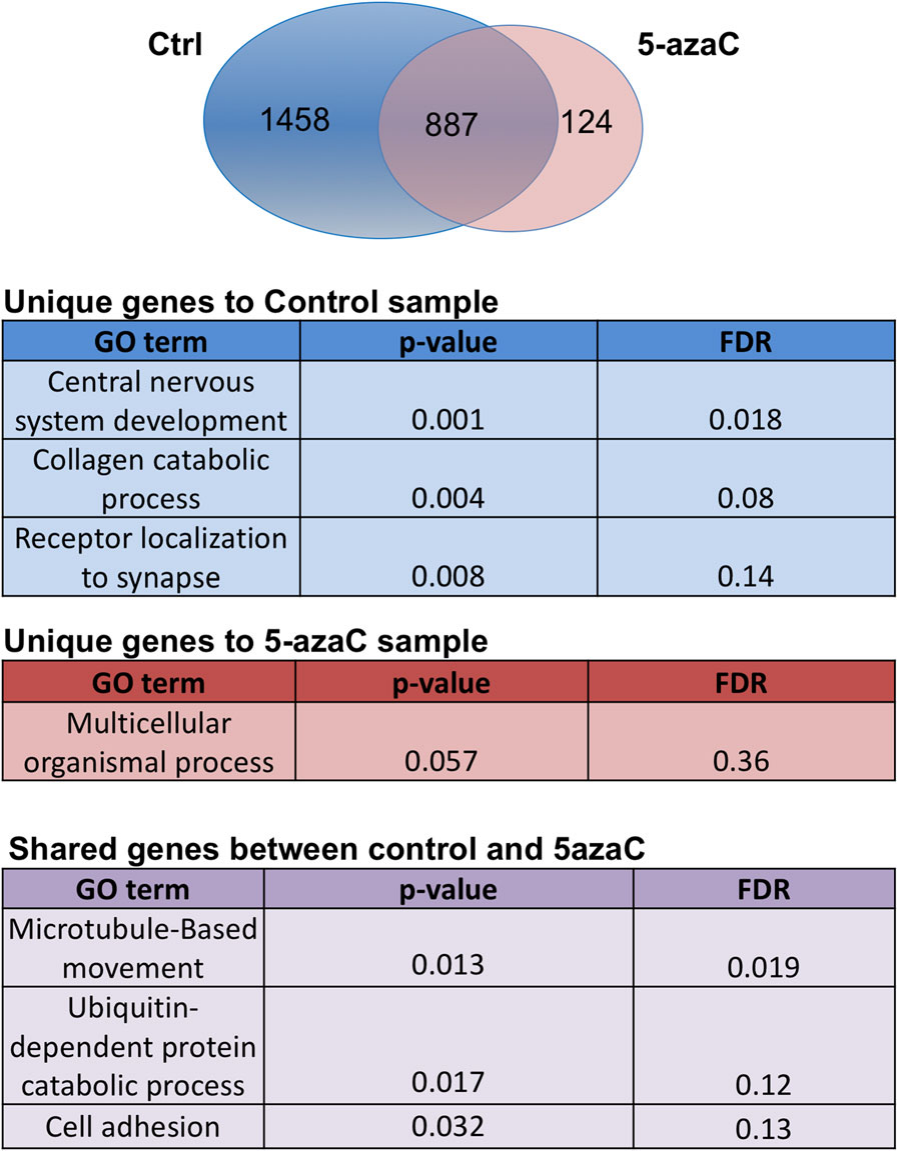
Target genes and gene ontology (GO) associated with SMCHD1 binding. (Top) Venn diagram illustrating number of unique target genes in control (ctrl) and 5-azaC treated samples as well as shared genes between two groups. (Bottom) GO terms associated with each sample group

Next, we sought to identify genes associated with SMCHD1 genomic binding. Using PAPST software, we assigned SMCHD1 binding peaks +/− 1750 Kb relative to transcriptional start site of genes. Using DAVID Bioinformatic Resources, we performed gene ontology analysis and identified biological processes associated with the selected genes. Out of 2345 genes associated with SMCHD1 binding in control cells, 1458 genes were sensitive to 5-azaC treatment that are significantly associated with central nervous system (Fig. 4, Additional file 3: Table S1). In addition, 124 novel target genes were identified following 5-azaC treatment that are associated with mRNA processing and regulation of cell growth. The list of gene ontology analysis is presented in Additional file 4: Table S2.

In an attempt to understand the nature of SMCHD1 DNA binding sites, we examined the composition of DNA sequences from called peaks where SMCHD1 was likely bound in cells grown under normal conditions as well as in cells treated with 5-azaC. Under normal growth conditions, we found that SMCHD1 bound DNA sequences near binding sites for beta/beta/alpha-zinc fingers, helix-loop-helix families, hormone receptors and homeodomain proteins. A summary of the frequency of the binding sites for these and other transcription factors are illustrated in Additional file 1: Figure S1, including the consensus motifs and the associated transcription factor with the highest Z scores from the most frequently occurring transcription factor families. A total of 128 motif clusters were identified in peaks obtained from cells cultured under normal conditions and only 6 in cells treated with 5-azaC. A complete list of individual transcription factors is provided in the supplemental information (Additional file 5: Table S3).

### SMCHD1 regulates *KCNQ1* gene expression

**Fig. 5:**
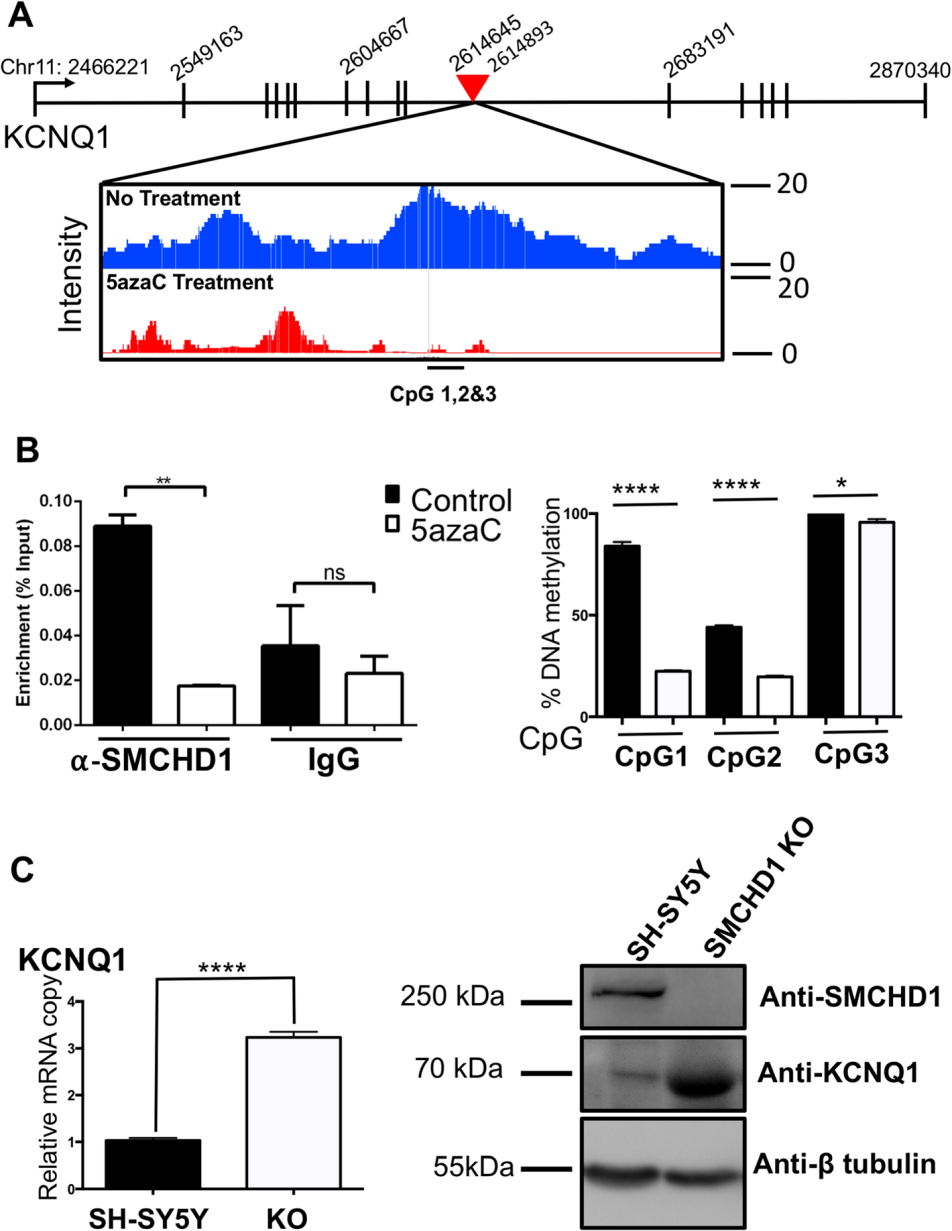
SMCHD1 occupancy over the *KCNQ1* intronic region is sensitive to 5-azaC treatment. **a** Schematic illustration representing SMCHD1 binding peaks in SH-SY5Y vehicle treated cells (blue) and cells treated with 5-azaC (red) over an intronic region of *KCNQ1* gene. The red triangle indicates the region examined in Fig. 5B by ChIP-PCR. **b** Left, ChIP-PCR of SMCHD1 in control (vehicle treated) and 5-azaC treated samples over *KCNQ1* intronic region. Right, percent DNA methylation of CpG sites located within SMCHD1 binding sites following 5-azaC treatment, CpG1: chr11: 2,614,727. CpG2: chr11:2,614,759. CpG 3 chr11:2,614,798. **c** Left, mRNA quantification of *KCNQ1* in SH-SY5Y (ctrl) and SMCHD1 KO cells (KO). The copy numbers are relative to β-actin cDNA levels. Right, SMCHD1 and KCNQ1 protein levels following SMCHD1 KO in SY-SY5Y cells. β-tubulin was used as an internal control

In order to validate the role of DNA-methylation in the transcriptional function of SMCHD1, we selected *KCNQ1* from the gene set associated with central nervous system. Previously, we and others showed that SMCHD1 regulates expression of the *KCNQ1* gene [1–3]. Here, the ChIP-seq data suggests potential occupancy of SMCHD1 located within an intronic region of *KCNQ1* gene (Fig. 5a). The ChIP-PCR confirmed SMCHD1 binding over this region in cells with no treatment, while SMCHD1 binding was significantly reduced in 5-azaC treated samples (Fig. 5b, left panel). In addition, 5-azaC treatment significantly lowered methylation level of CpG sites positioned within SMCHD1 binding region (Fig. 5b, right panel). Next, to examine the role of SMCHD1 in regulating *KCNQ1* gene expression, using CRISPR-cas 9, we knocked out SMCHD1 in SH-SY5Y cells. Our data suggests that *KCNQ1* mRNA and protein levels were both elevated upon SMCHD1 KO in SH-SY5Y cells (Fig. 5c).

## Discussion

This study provides a high-resolution mapping of SMCHD1 genomic occupancy sites that are sensitive to DNA demethylating reagent, 5-azaC. Consistent with a previous study in murine neuronal stem (NSC) cells which showed that SMCHD1 occupancy is not restricted to gene promoters [20], we found that SMCHD1 more actively binds to introns and intergenic regions in a 5-azaC sensitive manner in human neuroblastoma SH-SY5Y cells. In line with current findings in individuals with FSHD2, where the *DUX4* gene is hypomethylated with reduced binding by SMCHD1 [7], our data shows that in SH-SY5Y cells, SMCHD1 occupies D4Z4 repeats surrounding the *DUX4* gene and its binding is significantly reduced by 5-azaC treatment (Fig. 2a). Here, we also showed that loss of SMCHD1 increases *DUX4* gene mRNA accumulation inferring that SMCHD1 represses *DUX4* expression (Fig. 2c). There are two forms of FSHD, FSHD1 and FSHD2 [21]. While FSHD2 has a normal number of D4Z4 repeats, it harbors mutations in *SMCHD1* gene that correlates with hypomethylation of *DUX4*. In FSHD1, the more frequent form of FSHD, the number of D4Z4 repeats is reduced, which corresponds with loss of DNA methylation. DUX4 encodes a homeodomain protein which usually silent when there are more D4Z4 sets of repeats and is typically only expressed in germline. These individuals might harbor a *SMCHD1* mutation(s) that amplifies the severity of symptoms suggesting that *SMCHD1* mutation can act as a modifier of the disease [8]. These findings suggest that regulation of this region under SMCHD1 control might be through both DNA methylation-dependent and independent pathways. Indeed, the nature of SMCHD1 binding to this region and its role in regulating expression of this region requires further investigation.

SMCHD1 binding sites overlap with specific transcription factor binding motifs. A previous study suggested that SMCHD1 action may oppose that of CTCF in murine NSCs [20] . Our ChIP-seq data analysis and motif search suggested the CTCF binding motif as one of the SMCHD1 binding sites (Additional file 5: Table S3). However, this motif was not the most common in SMCHD1 ChIP-seq peaks (number 93 in the list of ordered motifs based on *p*-value). In our study, DNA motifs identified at high frequency in SMCHD1 ChIP-seq peaks include beta/beta/alpha-zinc fingers, helix-loop-helix families, hormone receptors and homeodomain proteins. The majority of DNA binding motifs were sensitive to 5-azaC treatment with only a limited number that were refractory to 5-azaC. These include beta/beta/alpha-zinc-finger proteins, nuclear hormone receptors, Rel Homology region proteins and the CENP-B box binding family. The beta/beta/alpha-zinc-finger motif was the most common zinc finger motif that acts as a DNA binding domain and is found in various transcription factors including GLI3 [22]. GLI3 acts as both an activator and repressor of the Sonic hedgehog (Shh) signaling pathway [22]. The GLI3 DNA binding domain has the lowest p-value (8.36E-70) in SMCHD1 binding motifs (Additional file 5: Table S3). Interestingly, our ChIP-seq analysis also associates SMCHD1 binding peaks with *GLI3* gene in a 5-azaC sensitive manner (Additional file 3: Table S1). Further investigation is required to determine SMCHD1 role in regulating *GLI3* gene expression and its involvement in axon guidance and development. The second most abundant motifs belong to RXR-gamma and NR2F6 from the hormone nuclear receptor family. RXR-gamma belongs to the retinoid X receptor (RXR) family of nuclear receptors that mediate the effects of retinoic acid (RA), and NR2F6 acts as a transcriptional repressor [23]. The significance of SMCHD1 binding to these DNA binding motifs and whether SMCHD1 has similar or opposing function requires further investigation.

We used gene ontology and functional annotation bioinformatics to determine biological processes that associate with SMCHD1 genomic binding and if they are affected by 5-azaC treatment. SMCHD1 genomic occupancy greatly influenced by 5-azaC treatment and the majority of binding sites were lost. However, there were binding sites that were not affected by 5-azaC treatment. GO analysis suggests that these genes are involved in microtubule-based movement. In addition, SMCHD1 occupied genomic regions unique to 5-azaC treated samples that are associated with mRNA processing suggesting that SMCHD1 might have gained new binding sites when DNA methylation level is reduced. These findings suggest that CpG demethylation may unmask SMCHD1 binding sites that regulate gene expression. However, we cannot discount the possibility that this may have arisen as a cellular response to 5-azaC toxicity or non-specific binding of SMCHD1 following loss of DNA methylation marks.

Previous data suggest that SMCHD1 might act to establish and/or maintain repressive chromatin marks. SMCHD1 mutation in FSHD2 individuals results in a dramatic loss of DNA methylation at several autosomal regions not limited to *DUX4* repeats [7]. In addition, SMCHD1 regulates gene clusters subjected to monoallelic expression and loss of SMCHD1 results in significant loss of DNA methylation [1–3]. We previously isolated SMCHD1 using methylated DNA in an affinity purification column. Here, our data introduce the possibility that DNA methylation is required for recruitment and binding of SMCHD1 to chromatin. Previous experiments in embryonic stem cells supported the role of SMCHD1 in late stage methylation and repression during X chromosome inactivation, whereas the initial DNA methylation marks appear to be independent of SMCHD1 function [5]. Whether initial methylation marks are necessary for guiding SMCHD1 to target regions for further establishment and/or maintenance of chromatin repression is unknown. We also found 887 target genes that were not affected by 5-azaC treatment. Whether SMCHD1 binding to these regions was independent of DNA methylation if 5-azaC treatment was ineffective remains unknown. Recent advances in molecular structure and function of SMCHD1 propose that SMCHD1 hinge domain is required for its interaction with chromatin and silencing of target regions [18]. In addition, SMCHD1 mutations lead to very different developmental diseases, FSHD and BAMS [24]. Unlike mutations in FSHD that are distributed along different domains of SMCHD1, all mutations identified in BAMS are located in the ATPase domain. SMCHD1 mutations holds a complex relationship with these very different developmental diseases as FSHD individuals do not present facial abnormalities observed in BAMS and BAMS individuals do not display muscular dystrophy [24]. So far, only one common mutation has been identified in both FSHD and BAMS, however individuals carrying this mutation do not display both symptoms [10]. Nonetheless, these findings suggest that disrupting the binding properties of SMCHD1 to other proteins or DNA could result in very different outcomes. In addition, it would be interesting to examine if mutation in the hinge domain region affects SMCHD1 genomic occupancy and whether DNA methylation status of chromatin modifies specificity and sensitivity of the SMCHD1 hinge domain binding to chromatin.

From SMCHD1 target regions, we chose to focus on ones that were sensitive to 5-azaC treatment, specifically the *KCNQ1* gene. We previously showed that SMCHD1 regulates expression of an imprinted gene cluster that overlaps with *KCNQ1*. KCNQ1 is a subunit of the voltage gated potassium channel, Iks, which mediates the slow delayed rectifying potassium current and crucial for cardiac action potential repolarization [25–27]. Co-assembly of KCNQ1 and another member of the KCN family of proteins like KCNE1 generates the Iks K^+^ current. Mutations in the *KCNQ1* and *KCNE1* interface cause long QT syndrome and atrial fibrillation which results in prolongation of the QT interval of heart repolarization [28, 29]. The ChIP-seq data suggests potential occupancy of SMCHD1 in intronic regions of the *KCNQ1* gene which is lost upon 5-azaC treatment. In addition, loss of SMCHD1 results in upregulation of *KCNQ1* at the mRNA and protein level. *KCNQ1* gene has been shown to be imprinted and monoallelically expressed. However, Sanger sequencing suggests that *KCNQ1* is not imprinted in SH-SY5Y cells and elevated levels of *KCNQ1* gene expression is due to up-regulation of both alleles (Additional file 2: Figure S2). To associate SMCHD1 function with regulation of imprinting and monoallelic gene expression, one would require a more suitable model such as human induced pluripotent stem cells that are known to maintain imprinting marks throughout development [30]. Our data suggests no other SMCHD1 occupancy near *KCNQ1* gene. It is possible that SMCHD1 regulates *KCNQ1* gene expression by mediating long-ranged chromatin interactions, as its role as a chromatin modifier have been suggested by other groups [31–34]. However, it is conceivable that SMCHD1 might indirectly regulate *KCNQ1* gene expression through regulating expression of transcription factors or other regulatory proteins. In addition, further investigation will be necessary to determine whether SMCHD1 role in mediating long-ranged chromatin interaction is facilitated by DNA methylation marks.

## Conclusion

In summary, we characterized SMCHD1 genomic binding sites and identified target regions that are sensitive to DNA demethylating reagent, 5-azaC. These results expand our knowledge on DNA methylation directed role of SMCHD1 as a chromatin modifier. Focusing on SMCHD1 target genes that are sensitive to DNA methylation, our data provides insight into the possible role of SMCHD1 in central nervous system development. From genes involved in central nervous system development, we specifically demonstrated DNA methylation sensitive genomic occupancy of SMCHD1 to KCNQ1 gene, a known target of SMCHD1. Future experimental strategies are necessary to decipher SMCHD1 molecular function in orchestrating regulation of genes involved in central nervous system, which will greatly impact future drug development.

## Methods

### Cells, antibodies and reagents

The cell line used in this study was SH-SY5Y (ATCC, CRL-2266). 5-Azacytidine (5-azaC) was purchased from Sigma (A1287). Antibodies used in this study included, anti-β-tubulin (Abcam, ab6046), commercial anti-SMCHD1 antibody (Bethyl, NBP1–49969), anti-SMCHD1 antiserum (produced in house), anti-LSD1 antiserum (produced in house), anti-DNMT1 (Abcam, ab19905).

### Cell culture and 5-azaC treatment

SH-SY5Y cells were cultured in Dulbecco’s Modified Eagle’s Medium (DMEM; Gibco) containing 4.5 g/L Glucose and L-Glutamine (Bio Whittaker, Cat. # 12-604F) which was supplemented with 10% fetal bovine serum (FBS). The cells were maintained in a humidified atmosphere which contained 5% CO2 at 37 °C. For 5-azaC treatment, cells were treated with 10 μM 5-azaC every 24 h for a period of 72 h to induce global loss of DNA methylation.

### SDS-PAGE and immunoblot

For preparation of whole cell lysate, cells were pelleted and washed once with PBS, then lysed in lysis buffer (PBS containing 1% triton X-100). Following resuspension of the pellet, the cells were sonicated briefly (Branson Sonifier 450. output 3.5 and constant duty cycle in pulses) and incubated on ice for 20 min, vortexed and then centrifuged for 5 min at 14,000 g. The supernatant was quantified, diluted and boiled in sample buffer for 5 min. Proteins were separated on 6% SDS-PAGE acrylamide gels using Tris-glycine buffering system [35]. After transferring of gels to PVDF membranes, the membranes were blocked in 0.05% milk powder in PBS containing 0.01% Tween-20 and then incubated with primary antibody (1:1000 dilution) overnight. Washes were done using PBS + 0.01% Tween-20, and then the membranes were incubated with secondary HRP antibody (Jackson Labs,1: 50,000 dilution). The membranes were developed using SuperSignal West Dura Extended Duration Substrate (Thermo Scientific, Cat. # 37071) and a cooled CCD instrument (Dyversity, Syngene) was used for detection.

### ChIP-seq antiserum development

We designed immunogenic SMCHD1 peptides to generate anti-SMCHD1 antiserum from guinea pigs and used this antiserum for ChIP-seq. Nucleotides encoding amino acids 1620–1727 of human SMCHD1 were expressed in *E. coli* BL21(pLsyS) using the PET28a expression system (Novagen). The peptide antigen was isolated on a Nickel column using standard denaturing conditions (guanidium hydrochloride /Urea, Qiaexpressionist, Qiagen). After elution, the denatured peptide was renatured using a stepwise dilution protocol until the final buffer contained PBS. Next the peptide was mixed with alum for inoculation into a guinea pig. Following a standard 90-day inoculation protocol with a number of boosts, blood was collected, and the serum tested for its efficiency in immunoprecipitation and used for ChIP seq.

### ChIP-seq assay

We examined the ability of our ChIP-seq anti-SMCHD1 antiserum to immunoprecipitate SMCHD1 compared to a commercially available anti-SMCHD1 antibody. Chromatin immunoprecipitation with 5-azaC treated and control SH-SY5Y cells was performed as previously described [2]. Briefly, twenty replicates (10 cm plates) of 5-azaC treated and control (vehicle treated) SH-SY5Y cells were fixed using 1% formaldehyde in HEPES (pH 7.8) for 8 min at room temperatures. Cells were then washed with PBS and collected. Equal cell volume of control and 5-azaC treated samples were re-suspended in lysis buffer (50 mM Tris-HCl (pH 8.1), 1% SDS and 10 mM EDTA) and sonicated using a Branon Sonifier 450 with an output of 3.5 and constant duty cycle in pulses to obtain 100–300 bp cross-linked DNA fragments. Five percent of the fragmented cross-linked chromatin was used as input and the rest was incubated with either 40 μL of anti-SMCHD1 antiserum or guinea pig serum overnight at 4 °C. Next, Protein A Sepharose beads were added 20 min prior to washing. The beads were then washed with RIPA (10 mM Tris-HCl pH 8.0, 1 mM EDTA, 0.5 mM EGTA, 140 mM NaCl, 1% Triton-X 100, 0.1% sodium deoxycholate, 0.1% SDS and 1X protease inhibitor cocktail (Bioshop, Cat. # PIC003)), TSEII (20 mM Tris-HCl pH 8.1, 500 mM NaCl, 2 mM EDTA, 0.1% SDS, 1% Triton X-100), TSE III (10 mM Tris-HCl pH 8.1, 0.25 M LiCl, 1 mM EDTA, 1%NP-40, 1% sodium deoxycholate) buffer and then 3 washes with 0.1X TE. DNA crosslinks were reversed with 0.1 M NaHCO3 and incubated overnight at 65 °C. The replicate DNA samples were pooled and DNA was precipitated using 2 μL of Pellet Paint (Novagen), 1/10 volume 3 M Na-acetate and 2 volumes of 100% EtOH by centrifugation for 10 min at 14,000 rpm. DNA pellets were washed with 70% EtOH, dried and re-suspended in 50 μL ddH2O. Michael Smith Genome Science Centre, Vancouver, Canada performed the sequencing. The DNA libraries were prepared according to the Illumina’s (2000/2500) suggested protocol followed by paired-end sequencing. Details are available from their website http://www.bcgsc.ca/platform/solexa/.

### ChIP-seq data analysis

To identify genomic sites bound by SMCHD1 with high resolution, we first mapped the reads to the human genome (GRCh37, hg19) (bam file, performed by Canada’s Michael Smith Genome Science Centre), then used MACS2 paired peak calling for identification of SMCHD1 peaks [36]. The *P*-value and q-value cutoffs were both set as 0.05. The raw sequencing files were submitted to NCBI (GEO number GSE99227). A total of 5051 peaks were identified for the control SH-SY5Y cells and 2100 peaks for the 5-azaC treated SH-SY5Y cells (BED file). For evaluating SMCHD1 peak positions relative to the transcriptional start sites (TSSs), the Genomic Regions of Enrichment Analysis Tool (GREAT) software and SeqMonk program [37] (available at http://www.bioinformatics.babraham.ac.uk/projects/seqmonk/) were applied. For motif analysis the SMCHD1 peaks were submitted to the SeqPos motif tool available in Galaxy cistrome [38]. Both cistrome and de novo motif search databases were used. The P-value cutoff was set as 0.001.

For assigning SMCHD1 peaks to associated genes, PAPST software was used [39] . Using the SMCHD1 peaks (BED file) obtained from MACS2 paired, peaks were assigned +/− 1750 kb relative to the TSSs of genes. The assigned genes were then submitted to DAVID Bioinformatics Resources 6.8 for identification of biological processes associated with SMCHD1 in control and 5-azaC-treated samples (Additional file 4: Table S2).

### CRISPR knockout of SMCHD1 in cells

We designed single guided RNA (sgRNA) targeting SMCHD1 and cloned them into the CRISPR-Cas9 PX459 plasmid following the Zhang Lab protocol [40] . In brief sgRNAs were designed using http://crispr.mit.edu/ software (Additional file 6: Table S4). The SMCHD1 sgRNA was designed to target exon 18 of SMCHD1. The sgRNAs were then cloned into CRISPR-Cas9 PX459 plasmid using BbsI according to the Zhang Lab protocol. Clones were sequenced (using oligonucleotide: gagggcctatttcccatgattcc) for confirmation of a positive clone. Transfection of SH-SY5Y cells was performed using the jetPRIME transfection reagent (VWR Cat# CA89129–922) according to the manufacturer’s protocol. Stably transfected cells were selected using 3μg/ml puromycin 48 h upon transfection.

### Reverse transcription quantitative PCR

For RNA extraction, Trizol (Life Technologies, Cat. # 15596018) was used according to the manufacturer’s protocol. About 200 ng of RNA was reverse-transcribed using Superscript II (Life Technologies, Cat. # 18964–014). StepOne Real Time PCR System (Life Technologies) and SYBR Advantage qPCR Premix (Clontech, Cat. # 638320) was used for quantification of cDNA. The oligonucleotides used in this work are listed in Additional file 6: Table S4. Following PCR, the PCR products were run on an agarose gel for confirmation of a single band amplification at the expected size. The threshold levels of each amplification were adjusted to the logarithmic part of the curve for determining a Ct value. Then the Ct values were normalized with those of β-actin to obtain the relative mRNA levels. The normalized data were analyzed using a Student’s t-test, and the confidence levels were displayed as *p*-values.

### Bisulfite pyrosequencing

Genomic DNA of SH-SY5Y control and KO cells were prepared using the Qiagen Blood and Cell culture kit (Qiagen, Cat. # 13323). The samples were treated with bisulfite using the Imprint DNA Modification Kit (Sigma, Cat. # MOD50-1KT). DNA samples were amplified by PCR. PCR primers were designed using the PyroMark Assay Design software 2.0 from Qaigen. PCR products were bound to streptavidin Sepharose beads (GE Healthcare Cat. # 17–5113-01), 10 μL of samples were sequenced using PyroMark Q24 pyrosequencer. Percent DNA methylation was then measured for each CpG site.

## Supplementary information

**Additional file 1: Figure S1.** Types of transcription factor (TF) family binding sites found associated with SMCHD1 binding.

**Additional file 2: Figure S2.** KNCQ1 is expressed bi-allelically in SH-SY5Y cells.

**Additional file 3: Table S1.** Genes associated with SMCHD1 genomic occupancy.

**Additional file 4: Table S2.** Ontology analysis of genes associated with SMCHD1 genomic occupancy.

**Additional file 5: Table S3.** Motif clusters associated with SMCHD1 genomic occupancy.

**Additional file 6: Table S4.** List of Primers.

## Data Availability

The ChIP-seq datasets generated in this study are available in the GEO repository number GSE99227.
