# Supplementary figures and images for: The effects of the DNA Demethylating reagent, 5-azacytidine on SMCHD1 genomic localization
Source: BMC Genet. 2020 Jan 15;21:3. doi: 10.1186/s12863-020-0809-x (PMC6964063; doi:10.1186/s12863-020-0809-x)

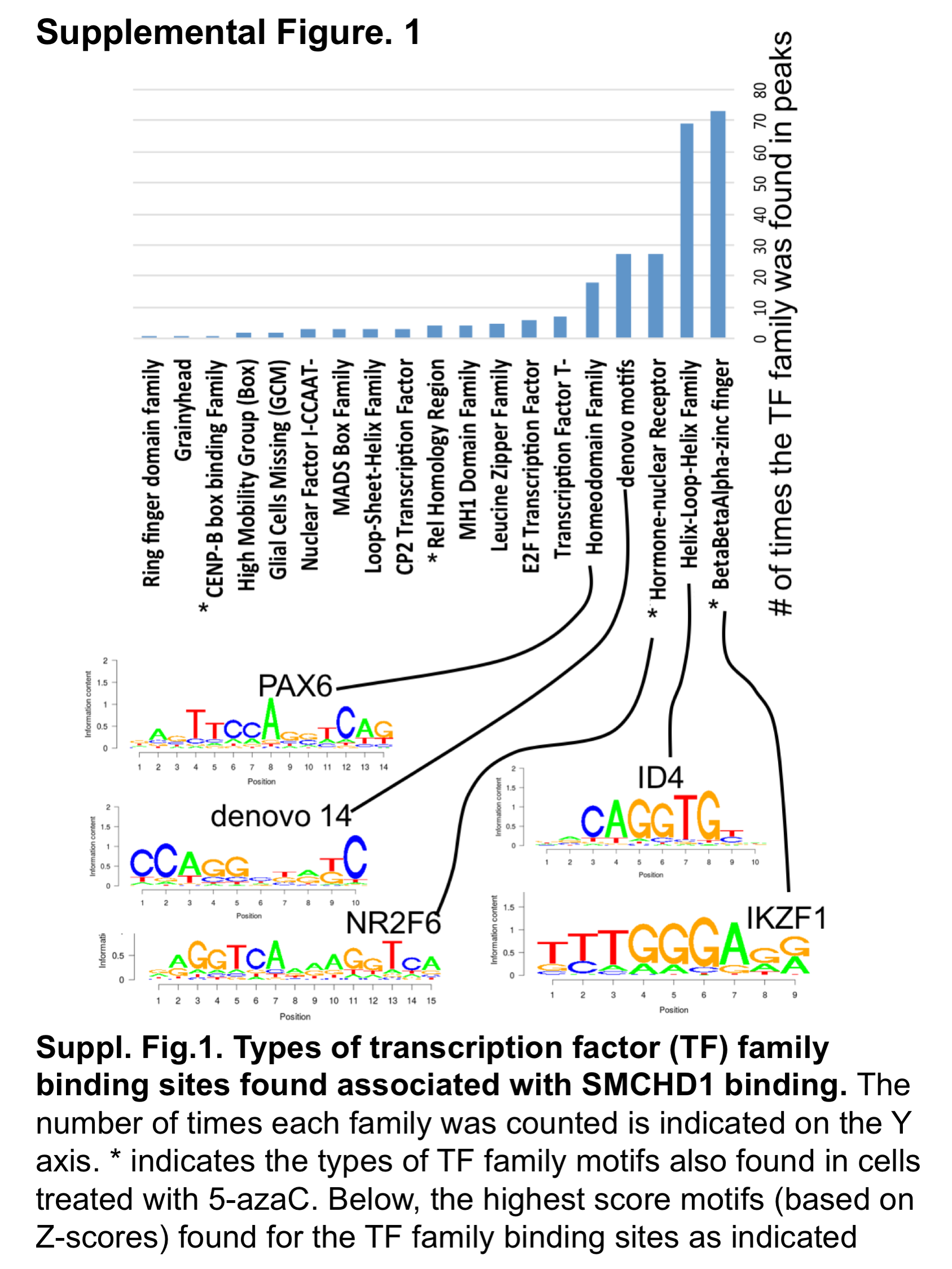

Supplement: Supplementary file 1 — Additional file 1: Figure S1. Types of transcription factor (TF) family binding sites found associated with SMCHD1 binding. [file 12863_2020_809_MOESM1_ESM.tiff]

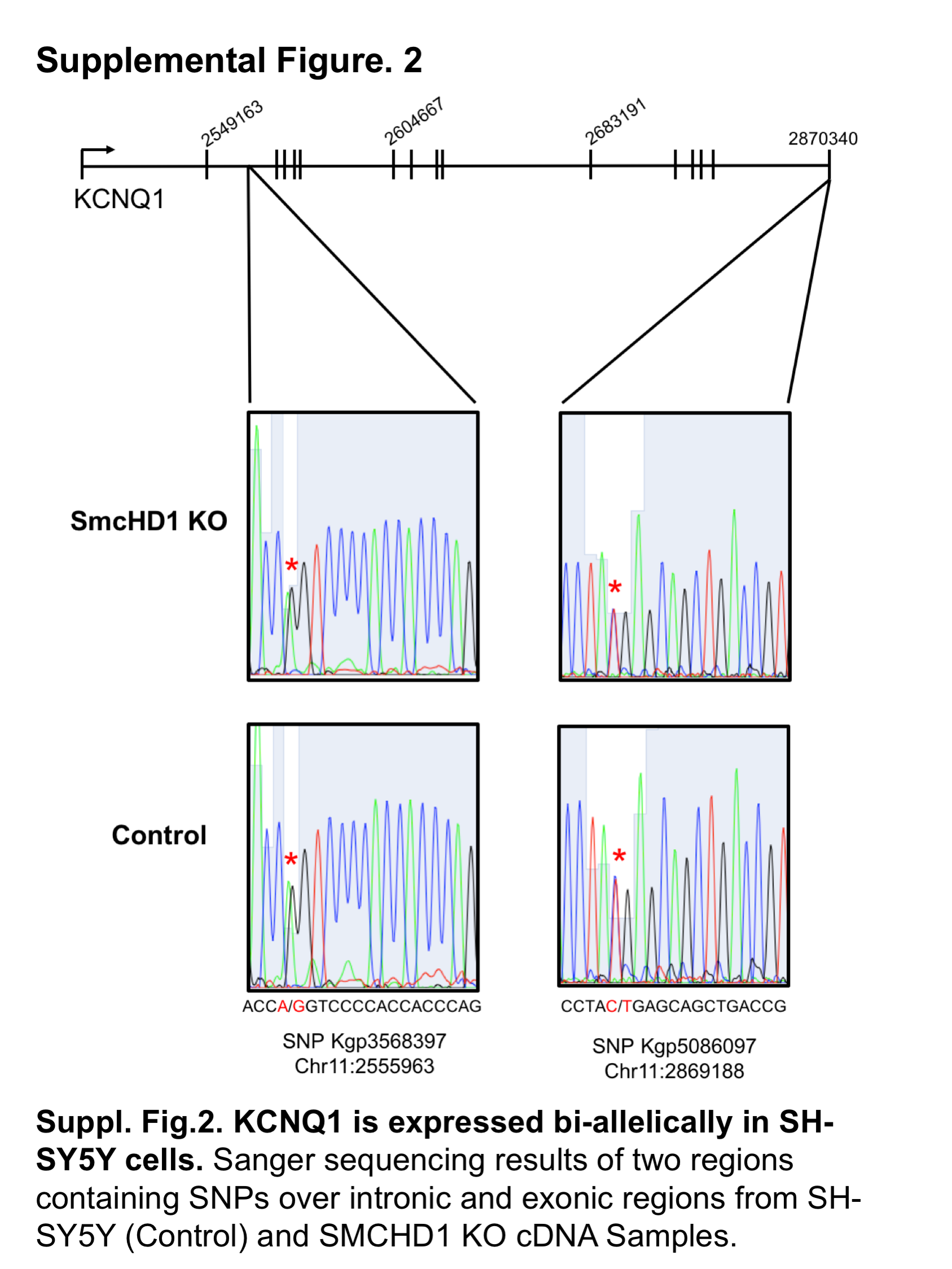

Supplement: Supplementary file 2 — Additional file 2: Figure S2. KNCQ1 is expressed bi-allelically in SH-SY5Y cells. [file 12863_2020_809_MOESM2_ESM.tiff]
